# Integration of Metabolomics and Transcriptomicsto Comprehensively Evaluate the Metabolic Effects of *Gelsemium elegans* on Pigs

**DOI:** 10.3390/ani11051192

**Published:** 2021-04-21

**Authors:** Chong-Yin Huang, Kun Yang, Jun-Jie Cao, Zi-Yuan Wang, Yong Wu, Zhi-Liang Sun, Zhao-Ying Liu

**Affiliations:** 1 College of Veterinary Medicine, Hunan Agricultural University, Changsha 410128, China; huangchongyin@163.com (C.-Y.H.); yangkun409@163.com (K.Y.); gquwi@Sina.cn (J.-J.C.); 15874806252@163.com (Z.-Y.W.); wuyong712@126.com (Y.W.); sunzhiliang1965@aliyun.com (Z.-L.S.); 2 Hunan Engineering Technology Research Center of Veterinary Drugs, Hunan Agricultural University, Changsha 410128, China

**Keywords:** *Gelsemium elegans*, metabolism, transcriptome, weaned piglet

## Abstract

**Simply Summary:**

Some natural phytogenic feed additives, which contain several active compounds, have been shown to be effective alternatives to traditional antibiotics. *Gelsemium elegans* (*G. elegans*) has been used as a traditional Chinese herbal medicine for many years, and it has an obvious growth-promoting effect on animals such as pigs. To our knowledge, the internal mechanism of the influence of *G. elegans* on the animal body is still unclear. Here, the plasma metabolomics and liver transcriptional profile of crude extract of *G. elegans* in pigs were reported for the first time and the metabolic consequences of feeding piglets *G. elegans* for 45 days were evaluated. The results showed that the addition of 2% *G. elegans* powder to feed is nontoxic to pigs. In addition, *G. elegans* could be used as a phytogenic feed additive, which could improve the immune function of piglets, and the latent mechanism of *G. elegans* may be related to various signaling pathways, including the MAPK signaling pathway and PPAR signaling pathway. Collectively, results of the current provide a clearer understanding of the molecular mechanism of the pharmacological effects of *G. elegans*, which is of great significance for a safer and more rational application of this phytogenic feed additives.

**Abstract:**

Some naturalphytogenic feed additives, which contain several active compounds, have been shown to be effective alternatives to traditional antibiotics. *Gelsemium elegans* (*G. elegans*) is a whole grass in the family Loganiaceae. It is a known toxic plant widely distributed in China and has been used as a traditional Chinese herbal medicine for many years to treat neuropathic pain, rheumatoid pain, inflammation, skin ulcers, and cancer. However, *G. elegans* not only is nontoxic to animals such as pigs and sheep but also has an obvious growth-promoting effect. To our knowledge, the internal mechanism of the influence of *G. elegans* on the animal body is still unclear. The goal of this work is to evaluate the metabolic consequences of feeding piglets *G. elegans* for 45 days based on the combination of transcriptomics and metabolomics. According to growth measurement and evaluation, compared with piglets fed a complete diet, adding 20 g/kg *G. elegans* powder to the basal diet of piglets significantly reduced the feed conversion ratio. Results of the liver transcriptome suggest that glycine and cysteine-related regulatory pathways, including the MAPK signaling pathway and the mTOR signaling pathway, were extensively altered in *G. elegans*-induced piglets. Plasma metabolomics identified 21 and 18 differential metabolites (*p* < 0.05) in the plasma of piglets in the positive and negative ion modes, respectively, between *G. elegans* exposure and complete diet groups. The concentrations of glycine and its derivatives and N-acetylcysteine were higher in the *G. elegans* exposure group than in the complete diet group.This study demonstrated that *G. elegans* could be an alternative to antibiotics that improves the immune function of piglets, and the latent mechanism of *G. elegans* may be related to various signaling pathways, including the MAPK signaling pathway and the PPAR signaling pathway.

## 1. Introduction

In the current trend of “restricted feeding antibiotics” or even “no feeding antibiotics” in livestock and poultry production, phytogenic feed additives (PFAs), as plant-derived products, are often used in animal feed to improve the performance of livestock. PFAs have complex compositions and complex mechanisms of action. Thus, the study of the mechanisms of PFAs is important in the rational and scientific development of PFAs for livestock.

*Gelsemium elegans* (*G. elegans*), one of three species of *Gelsemium*, is a genus of flowering plants belonging to the family Loganiaceae [1]. *G. elegans* is mainly distributed in Southeast Asia and has been used as a traditional Chinese medicine for treating neuropathic pain, rheumatoid pain, spasms, skin ulcers, and cancer for many years [2,3]. *G. elegans* is a known toxic plant, and its toxicity limits its appropriate dosage and clinical use. However, interestingly, *G. elegans* is not toxic to pigs, sheep, and other animals and has an obvious growth-promoting effect. Liu et al. discovered and reported in 1994 that the average weight of pigs fed a diet with *G. elegans* was 11.6 kg higher than that of pigs fed a diet without *G. elegans*, and the weight gain-promoting effect of *G. elegans* was obvious [4]. The combined use of ginseng (with *G. elegans*) as a feed additive can increase the daily weight gain of pigs by 16.6% and the feed conversion rate by 18.2%, improving economic benefits for farmers [5].Chen et al. fed *G. elegans* extract to pigs for 49 day, and the average daily gain and feed intake of pigs was improved significantly (*p* < 0.05), and the feed conversion ratio was reduced significantly (*p* < 0.05) [6]. All these results suggest that *G. elegans* has a good effect on promoting animal growth. At present, Cao et al. found that gelsedine-type alkaloids were the major active ingredients that predict and explain the efficacy and toxicity of *G. elegans* [7]. However, the underlying mechanism of the influence of *G. elegans* on the animal body is still unclear.

The absence of an appropriate research method makes it difficult to clarify the mechanisms of most PFAs. However, the advent of omics technologies has made these analyses possible. Metabolomics is an important branch of systems biology. It is based on the analysis of the endogenous metabolites of various biofluids and tissue extracts and aims to identify latent relationships between changed metabolic profiles and the physiological status of the biosystem [8]. Consequently, it is reasonable to use metabolomics to explore the mechanism of PFAs and evaluate their safety. In contrast to metabolomics, transcriptomics studies gene expression, regulatory systems, and regulatory changes at the level of mRNA. By associating transcriptomics with metabolomics and by combining their respective advantages, a deeper truth of life can be revealed, and its wholeness can be known.

Recently, studies have shown that CYP3A4/5 in liver microsomes mediated the metabolism of *G. elegans* [9], and *G. elegans* has remarkable effects on lipid metabolism, such as attenuation of liver steatosis [10]. In addition, blood is the main carrier of oxygen, nutrient, and metabolite transport in the animal body. Largely unexplained effects of *G. elegans* were found using screening techniques, such as transcriptomics and metabolomics, on several other metabolic pathways, such as metabolism of amino acids and cellular stress response [11], overall indicating a multifaceted influence of *G. elegans* on metabolism. At present, there are few studies on the effect of *G. elegans* on pig liver and its molecular mechanism, and there are no metabolomic reports regarding the effects of *G. elegans* on pigs. Thus, in order to investigate the effects of *G. elegans* on the pigs’ metabolism as comprehensively as possible, several omics-techniques, such as transcriptomics and metabolomics, were applied on key metabolic tissues, such as liver and plasma of pigs. 

## 2. Materials and Methods

### 2.1. Materials and Reagents

Wild *G. elegans* were collected from Fujian province in China, which were collected during the vegetative period. Associate Professor Qi Tang at Hunan Agricultural University authenticated the samples. The samples were stored at our laboratory and the voucher number was No. 1537201809. The crude samples of *G. elegans* were dried and milled into a powder.

Methanol (MeOH), water (H_2_O), formic acid, and acetonitrile (ACN) were purchased from Thermo Fisher Scientific (Waltham, MA, USA), and the purity of the above reagents was LC–MS grade. AmpureBeads was purchased from Beckman Coulter Co. (Brea, CA, USA). Quant—iTPicoGreen dsDNA Assay Kit was purchased from Life Technologies (Carlsbad, CA, USA). TruSeq RNA LT Sample Prep Kit v2, TruSeq PE Cluster Kit v3-cBot-HS, TruSeq SBS Kit v3-HS (200-cycles) were provided by Illumina Co. (San Diego, CA, USA). Hunan Xinwufeng, Yong’an branch (Changsha, China) kindly provided the piglets.

### 2.2. Animals and Treatments

We used in the present study 20 healthy castrated male ternary hybrid piglets (initial BW = 20 ± 2 kg, 60 day old) fed a complete diet (the control group, *n* = 10) or a complete diet with 20 g/kg *G. elegans* powder (the *G. elegans*-treated group, *n* = 30). The formulations and nutrient levels of each diet have previously been reported [12]. The piglets were in a healthy condition and the living environment was provided in accordance with animal welfare standards in the whole experimental period. The experimental period lasted for 45d, and the weight and feed intake of piglets were recorded every two weeks. After the end of the experiment, the administration was stopped, and all piglets fasted for 24 h. The piglets were slaughtered within one day after stopping drug administration. Blood and urine were collected for clinical chemistry and hematology analyses. The ileum and liver were surgically removed and weighed. Details of the organization’s storage have been previously reported [13]. Briefly, a fraction of the livers were fixed with a 75% alcohol solution for histopathological examination; the remaining tissue sample was immediately placed in a centrifuge tube, sealed with a sealing membrane, quick-frozen in liquid nitrogen, and stored at −80 °C until the RNA was extracted from the tissue.

### 2.3. Plasma Biochemical and Immunity Parameters

A hematologic analyzer (Mindray bc—2800vet, Shenzhen, China) was used to analyze routine blood parameters such as red blood cells (RBS), hemoglobin (HGB), mean red blood cell hemoglobin (MCH) content, mean red blood cell hemoglobin concentration (MCHC), and white blood cell (WBC) count in whole blood. Liver tissues were embedded in paraffin, stained with hematoxylin and eosin, and examined under a light microscope (Aoxiang Optoelectronic UB103I, Chongqing, China).

### 2.4. Transcriptome Analysis

Livers from three pigs in each treatment group were randomly selected for transcriptomic analysis. RNA extraction and quality inspection were carried out on the sample tissue according to the TRIzol extraction kit instructions. After passing the quality inspection, double-ended (PE) sequencing was carried out to construct an RNA library using the Illumina HiSeq sequencing platform (Illumina, San Diego, CA, USA). The protocols were performed exactly as described in our previous publications [13].

Six important differentially expressed genes (DEGs) (ATP2B3, MAT2A, CHDH, SLC20A2, SLC28A1, MT3) were selected. Quantitative real-time PCR (qRT-PCR) verification was performed using a Tianlong TL988 fluorescence quantitative PCR instrument (Xi’an Tianlong Technology Limited). PCR data were analyzed using the comparative Ct (2^−ΔΔCt^) method.

### 2.5. Metabolomic Analysis

#### 2.5.1. Plasma Sample Collection and Preparation

Plasma samples (100 μL) were placed in 1.5 mL centrifuge tubes, and then 400 μL of an 80% methanol–water solution was added, vortexed, stored at −20 °C for 60 min, and centrifuged at 14,000 rpm and 4 °C for 20 min. Then, a certain amount of supernatant was placed in a 1.5 mL centrifuge tube, the mixture was lyophilized in vacuo, and the residue was reconstituted in 100 μL of solvent, vortexed, and centrifuged at 14,000 rpm and 4 °C for 15 min. The supernatant was then subjected to LC–MS/MS analysis.

#### 2.5.2. UPLC–MS/MS Conditions

Sufficient chromatographic separation was achieved with mobile phase A (positive ion mode: 0.1% formic acid–water, 95% acetonitrile, and 10 mM ammonium acetate; negative ion mode: 0.1% formic acid–water, 95% acetonitrile, and pH 9.0) and mobile phase B (positive ion mode: 0.1% formic acid–water, 50% acetonitrile, and 10 mM ammonium acetate; negative ion mode: 50% acetonitrile, 10 mM ammonium acetate, and pH 9.0). The gradient elution was as follows: 0–1.0 min, 98% A; 1–17.5 min, 50% A; and 17.5–20 min, 98% A. The sample injection volume was 20.0 μL. The MS/MS analytical conditions were as follows: The Turbo Spray source voltage and temperature were set to 3.2 kV and 320 °C, respectively.

#### 2.5.3. Metabolomics Data Processing

Raw data were collected using Thermo Scientific Exact Finder workstation software (Waltham, MA, USA), and the obtained data required preliminary screening by importing them into the Compound Discoverer (CD) database under conditions of retention time and *m*/*z* parameters. The XCMS software was for retention time correction, peak identification, peak extraction, peak integration, and peak alignment. The combination of supervised partial least squares-discriminant analysis (PLS-DA) and univariate statistical analysis was used to screen different metabolites. Qualitative analysis of metabolites was carried out by software-built secondary mass spectrometry database and a common database, such as mzCloud.

## 3. Results

### 3.1. Growth Performance and Routine Blood Analysis

Throughout this study period, no abnormality, no disease, and no death of piglets was observed. According to the growth measurement and evaluation, a week was selected for weighing and calculating the feed conversion ratio (Table 1). The results indicate that adding 20 g/kg *G. elegans* powder to the basal diet of piglets could significantly reduce the feed conversion ratio, but the feed intake was lower and the growth rate was lower as well. The results of routine blood examination (Table 2) suggest that there was no significant difference in most indexes between the *G. elegans*-treated and control groups (*p* > 0.05), but the number of neutrophils and leukocytes in the *G. elegans*-treated group increased. The mean corpuscular volume in the *G. elegans*-treated group was significantly lower than that in the control group (*p* < 0.05).

### 3.2. Analysis of the Liver Transcriptome under the Influence of G. elegans

**Figure 1:**
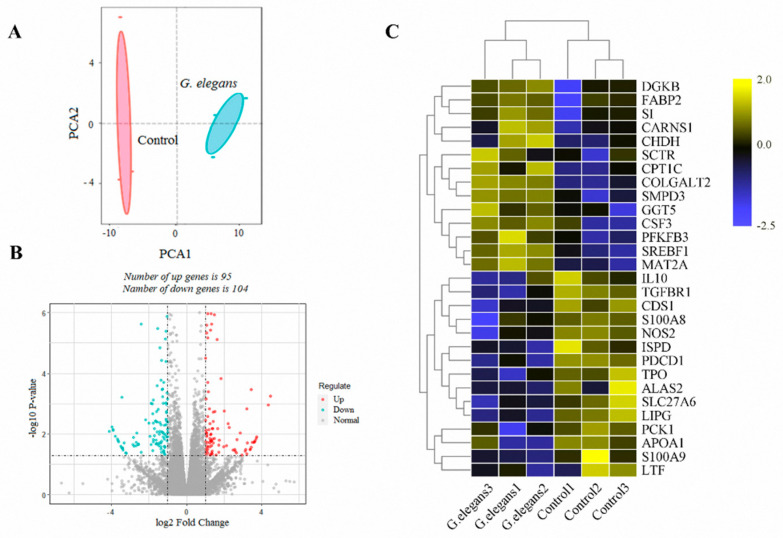
Differences in the expression levels of genes and metabolism-related alterations at the transcriptional level in piglet liver after *G. elegans* feeding. (**A**) PCA score plot of the genes identified from the control and *G. elegans*-treated group comparison. (**B**) Volcano plot of differentially expressed genes. Log2 fold changes in gene expression based on RNA-seq in the control and *G. elegans*-treated groups, and the corresponding significance values are displayed as log10 (*p* value). The transverse and vertical dotted lines indicate the cutoff value for differential expression (*p* < 0.05 and |log2 fold changes| > 1). In total, 95 and 104 genes with increased (red) or decreased (blue) expression levels induced by *G. elegans* exposure were identified. (**C**) Hierarchical clustering based on the DEGs related to metabolism.

**Figure 2:**
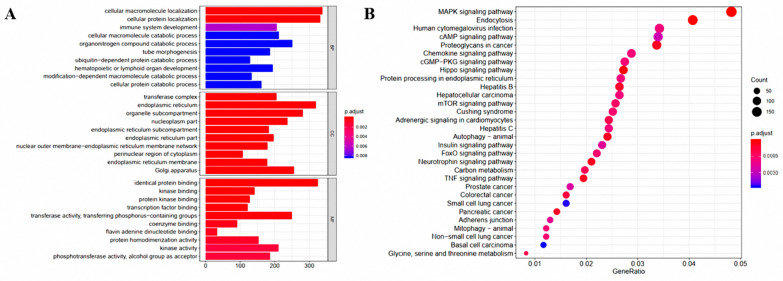
GO classification and pathway enrichment results of differentially expressed genes (DEGs) between liver tissues of the *G. elegans*-treated group and the control group. (**A**) GO classification of the DEGs. (**B**) Pathway enrichment results of the DEGs. The X axis represents the enrichment factor value, and the Y axis represents the pathway name. The color represents the corrected *p* value (*p* adjust < 0.05), and the size of the dots represents the number of genes. GeneRatio represents the proportion of enriched genes to background genes.

By analyzing the liver transcriptome of male piglets, we found a total of 199 DEGs, among which 95 were up-regulated and 104 were downregulated (Figure 1B). Subsequently, PCA was used to examine gene expression differences between groups. As shown in Figure 1A, the *G. elegans*-treated group was obviously separated from the control group by the first component (PCA1), and the results were consistent with the heat map. The differences in metabolism and immune-related genes according to the hierarchical clustering analysis heat map also indicate the differences between the two groups (Figure 1C). Results of RNA-seq analysis indicate that the expression levels of the lipid metabolism-related genes GGT5, SMPD3, CPT1C, FABP2, and DGKB; the carbohydrate metabolism-related genes SI and PFKFB3; and the amino acid metabolism-related genes MAT2A, CARNS1, COLGALT2, and GGT5 were all up-regulated; and the expression levels of immune-related regulatory genes S100A8 and S100A9 were significantly decreased. Analysis of the enriched GO (genetic ontology) terms among DEGs was performed to assess the effects of feeding *G. elegans* (Figure 2A). The most important terms in the biological processes (BP) category were “cellular protein localization” and “immune system development”. In the molecular function (MF) category, most catalytic gene binding functions (identical protein binding, kinase binding, protein kinase binding, transcription factor binding, etc.) were abundant, and the results suggest that the metabolic activities of animals were effectively activated by exposure to *G. elegans*. Functional enrichment analyses using the Kyoto Encyclopedia of Genes and Genomes (KEGG) pathways revealed the significant enrichment of several major metabolic pathways (Figure 2B). Glycine and cysteine-related regulatory pathways, including the MAPK signaling pathway and the mTOR signaling pathway, were extensively altered in *G. elegans*-treated piglets. Furthermore, the pathways associated with hepatitis B were significantly changed by exposure to *G. elegans*. Results of this study suggest that *G. elegans* induced changes in gene expression related to amino acid, lipid, and carbohydrate metabolism and immune regulatory pathways.

The ileum transcriptome results of male piglets were analyzed. The *G. elegans*-treated and control groups were compared and analyzed by using |log2 fold change| ≥ 1 and *p* ≤ 0.05 as the screening conditions. A total of 446 DEGs were identified in this study, of which 237 genes were up-regulated, and 209 genes were down-regulated. In general, a mixture of up-regulated and down-regulated DEGs was observed in the immune and inflammatory response pathways.

To verify the transcriptome analysis results of the liver and ileum, 6 important DEGs were selected for verification and quantification by qRT-PCR. As shown in Table 3, the results showed that the expression profiles of these genes as evidenced by qRT-PCR were consistent with those evidenced by the transcriptome analysis, which confirmed the reliability of our RNA sequencing data.

### 3.3. Analysis of the Plasma Metabolome under the Influence of G. elegans

**Figure 3:**
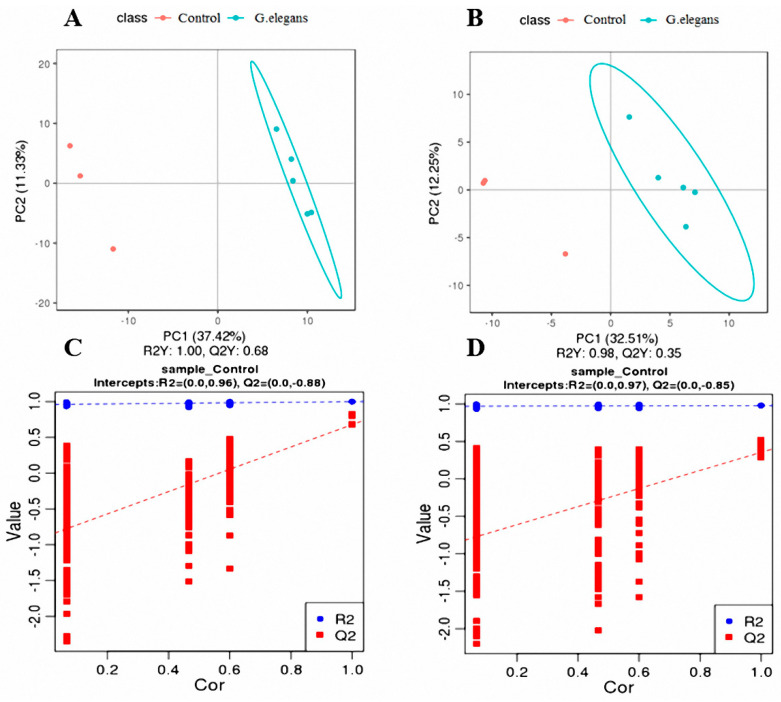
The PLS-DA for scatterplot and sorting verification plot in positive and negative mode. The scatterplot (**A**,**B**) was obtained. The abscissa is the score of the sample on the first principal component. The ordinate is the score of the sample on the second principal component. R2Y represents the interpretation rate of the second principal component of the model, and Q2Y represents the prediction rate of the model. In the sorting test (**C**,**D**), the abscissa represents the correlation between the random group Y and the original group Y, and the ordinate represents the score of R2 and Q2.

PLS-DA analysis was performed on all metabolites analyzed in the positive and negative ion modes to estimate the metabolic changes in piglets caused by different feeding conditions. In the positive ion mode, R2 = 0.96 and Q2 = 0.88, and in the negative ion mode, R2 = 0.97 and Q2 = 0.85, indicating that the model described the samples well and could be used to search for biomarkers in the next step. In the PLS-DA scatter plot (Figure 3), the *G. elegans*-treated and control groups were clearly separated, indicating that *G. elegans* could regulate normal metabolic pathways.

A total of 498 metabolites in positive ion mode and 293 metabolites in negative ion mode showed statistically significant differences, as evidenced by variable importance in the projection (VIP) value > 1.0 and *p*-value (*T*-test) < 0.05. Among these metabolites, 21 and 18 differential metabolites were identified in the plasma of piglets in the positive and negative ion modes, respectively, in the *G. elegans*-treated and control groups (Table 4 and Table 5).

### 3.4. Integrated Enrichment Analysis of Transcript and Metabolite Profiles

**Figure 4:**
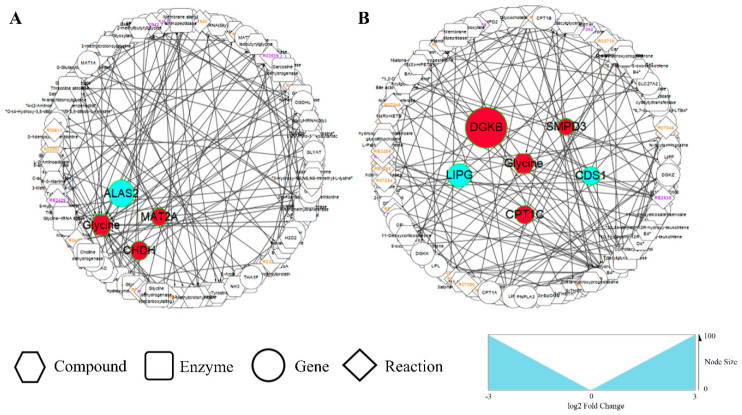
Network of genes and metabolites regulated by *G. elegans*. Network visualization of the amino acid metabolism (**A**) and lipid metabolism pathways (**B**) was performed using Cytoscape software (version 3.5.1) and MetScape software (version 3.1.3). The node colors in this figure correspond to absolute maximum log2 fold changes; significant increases and decreases in differential metabolites and differentially expressed genes are highlighted in red and blue, respectively.

Integrated enrichment analysis indicated that *G. elegans* supplementation could affect lipid metabolism, sugar metabolism, and amino acid metabolism, such as glutathione metabolism and glycine, serine, and threonine metabolism. Specifically, amino acid and lipid metabolic changes were mainly identified by the transcriptome and metabolome analyses (Figure 4), and sugar metabolism changes were mainly identified in the transcriptome analysis.

#### 3.4.1. Amino Acid Metabolism Alterations

Metabolomic and transcriptomic analysis confirmed the effect of *G. elegans* on amino acid metabolism. The most relevant pathways were glutathione metabolism and glycine, serine, and threonine metabolism. The increase in plasma glycine in piglets exposed to *G. elegans* may be related to bile acid synthesis and metabolism. Bile acid is an endogenous molecule synthesized by cholesterol in the liver. The liver combines bile acid with glycine or taurine in a two-step reaction, and then the combined bile acid is actively transported to bile and stored in the gallbladder. Until bile acid is released into the duodenum after eating, conjugated bile acid is reabsorbed into the ileum and circulated to the liver through the portal vein; this process is called enterohepatic circulation [14]. Results of the ileum transcriptomics analysis indicate that the expression level of the SLC51A gene was increased in the *G. elegans*-treated group, compared to the controls. Due to the induction of the ileum transporter encoded by this gene, the reabsorption of conjugated bile acid by the intestinal tract was enhanced [14], and the content of conjugated bile acid in the liver was increased through intestinal-hepatic circulation, which may lead to a decrease in the feedback of liver bile acid metabolism and a decrease in glycine binding reaction, leading to an upregulation of glycine in the plasma metabolite analysis.

N-acetylcysteine (NAC), the precursor of cysteine, is rapidly metabolized by the gut to generate GSH. Glycine can be converted into GSH by binding with l-glutamylcysteine. Although *G. elegans* exhibited no significant effect on the concentration of glutathione in the plasma of piglets, the metabolomic analysis results suggest that NAC and glycine derivatives were significantly up-regulated in the plasma of piglets exposed to *G. elegans*, compared to the controls. In addition, the expression level of the GGT5 gene encoding gamma-glutamyl transferase was up-regulated in the liver of piglets exposed to *G. elegans*.

#### 3.4.2. Lipid Metabolism Alterations

Results of transcriptomic analysis indicate that *G. elegans* induced a large number of changes in hepatic lipid metabolism genes in piglets. In the study, although *G. elegans* exhibit no significant effect on the lipid concentration in the plasma of piglets, koumine, an indole alkaloid isolated from *G. elegans*, significantly reduced the levels of triglycerides (TG), cholesterol (TC), low-density lipoprotein (LDL-C), alanine aminotransferase (ALT), and aspartate carbamoyl transferase (AST) in the serum of nonalcoholic fatty liver disease (NAFLD) rats [10] and increased the level of high-density lipoprotein (HDL-C). The expression level of the lipase gene associated hydrolyzed HDL-C, LIPG, was significantly lower in the livers of piglets exposed to *G. elegans* than in those of piglets fed the control diet. The expression levels of the sphingomyelin metabolic hydrolase-related gene SMPD3 and the lipid metabolism-related genes DGKB and CPT1C were up-regulated.

## 4. Discussion

### 4.1. Effect of G. elegans on Intake and Average Gain

Wu et al. [15] fed piglets with a basal diet containing 0.3% and 0.5% *G. elegans* powder for 30 days. The results showed that the weight gains of the experimental groups fed with 0.3% and 0.5% *G. elegans* powder were higher than those of the control group, and the feed conversion ratio was significantly reduced by 3.81% and 10.59%, respectively.

In this study, *G. elegans* could significantly reduce the material to weight ratio and promote the digestion and absorption of animals which is consistent with the previous report. However, contrary to the discovery of Wang et al. [16], the additional dose of 20 g/kg *G. elegans* whole grass did not significantly improve the growth of piglets, which might be due to the bitter taste of the *G. elegans* whole grass dosage form and poor palatability, which may affect the appetite of piglets. The whole grass dosage form should be avoided as much as possible, and the *G. elegans* extract form should be used for feeding in actual production and application. According to reports, at the dose of 50 mg/kg, *G. elegans* showed remarkable growth-promoting effects but had no significant effect on the average daily feed intake of piglets [17]. However, *G. elegans* has some kind of toxicity [18], so the residues in blood and muscle are a concern of people. It is reported that *Gelsemium* alkaloids are absorbed rapidly in pigs, and the T1/2 values of most *Gelsemium* alkaloids ranged from 8 h to 12 h, suggesting that the elimination was slow and there may still be residual levels in pigs [19]. Our team will further study the residue depletion of *G. elegans* in pigs for food safety.

### 4.2. Effect of G. elegans on Physiological Function of Weaned Piglets

Routine blood tests can effectively reflect the body’s resistance to diseases. The reduction of white blood cells and hemoglobin in the blood can be regarded as the signs of decreased antibody resistance and reactivity. T lymphocytes can not only mediate the cellular immune response of the animal body, but also, the cytokines secreted in the immune response have important regulatory effects on the immune response of the body, including the proliferation, differentiation, and function of immune cells.

Many studies reported that *G. elegans* alkaloids triggered the immune response by promoting the expression of pro-inflammatory factors [20] and affect the activation and proliferation of T lymphocytes [10]. The results of the present study showed that compared with the control group, the counts of neutrophils and leukocytes in the blood of the *G. elegans*-treated group increased, which indicates that *G. elegans* improved the cellular immune function and disease resistance of the weaned piglets. In addition, the mean red blood cell volumes in the *G. elegans-treated* pigs, although significantly reduced, remained within the normal reference range without adverse effects.

### 4.3. Evidence That G. elegans Regulates Amino Acid Metabolism and Decreases p38 Activation

In the study, the results of which are presented here, the metabolomics results emphasize a series of metabolites related to amino acids, including glycine and its derivatives and NAC, which suggests that the regulation of amino acid metabolism plays an important role in the immune stimulation of *G. elegans*. Glycine has recently been classified as a nutritionally essential amino acid for maximal growth in young pigs. If glycine is insufficient in the body, amino acid metabolism will be affected, resulting in intestinal dysfunction. Studies have shown that glycine can protect a variety of organs, such as the liver, skeletal muscle, and small intestine, from certain harmful substances [17]. Wang et al. [21] investigated the cellular protective effects of glycine and indicated that glycine stimulates protein synthesis in IPEC-1 cells and inhibits oxidative stress by increasing intracellular glutathione concentrations. Moreover, various studies have shown that glycine can produce anti-inflammatory effects by decreasing reactive oxygen species and inflammatory mediator levels [19], inhibiting inflammatory cell aggregation, and reducing lipid peroxidation [22]. NAC can protect intestinal health and has a therapeutic effect in the treatment of colitis [23] and hepatitis [24]. The effects of NAC have been reported to be associated with decreases in the proinflammatory cytokines TNF-α, IL1β, and IL-6 [23,25]. As *G. elegans* contains many active ingredients, it is difficult to directly attribute the specific metabolic effects of these active ingredients. However, drug effects and other exogenous stimulation always lead to variations in the metabolic network of endogenous metabolites, mainly reflected in the types and quantities of metabolites present. Metabolomics can be used to investigate the overall effect of stimulation on the body through comprehensive and systematic detection and analysis of endogenous small molecule metabolites in biological samples [26]. Therefore, there is no doubt that the discovery of amino acid-related metabolites may provide new insights into the mechanisms of the immune stimulation of *G. elegans*.

Results of the transcriptome analyses identified 199 DEGs in the liver, and through GO enrichment analysis, it was found that these transcripts were mainly enriched in the biological processes of proteins and amino acid metabolism and immune responses. Peptidyl-cysteinenitrosylation and peptidyl-cysteine modification were directly related to cysteine. Four of the down-regulated genes, including NOS2, LTF, S100A9, and S100A8, are well-known to be involved in multiple processes of cysteine regulation. NOS2 encodes a type of oxide synthase that is expressed in the liver and is inducible by a combination of lipopolysaccharide and certain cytokines. It mediates the nitrogenation of cysteine, participates in the inflammatory response, and enhances the production of NO and proinflammatory mediators such as IL-6 and IL-8 [27]. Thus, *G. elegans* can reduce the synthesis of proinflammatory mediators by regulating the expression of nitric oxide synthase. According to previous reports, koumine can inhibit the secretion of NO, ROS, TNF-α, IL-6, and IL-1β and significantly reduce the mRNA and protein levels of iNOS [1], which is consistent with our observations. S100A9 and S100A8 are both members of the S100 family of proteins. This family of proteins has a wide range of intracellular and extracellular functions and is considered an important regulator of macrophage inflammation, tissue damage, and regulatory stress [28]. Similarly, the decreased expression of LTF supports the anti-inflammatory effect, as the gene encoding lactoferrin is up-regulated during inflammation, activating the innate immune system through surface receptors, producing immune complexes containing LTF, and triggering the infiltration of monocytes and macrophages [29]. Consequently, results of the gene expression profile indicate that *G. elegans* can significantly regulate the expression of genes related to cysteine metabolism and achieve anti-inflammatory effects.

It is worth mentioning that our previous study reported the same batch of experimental ileum transcriptional spectrum data [12], and the results suggest that the inflammation-related genes (such as C3, C5, S100A8, IL-8/CXCL-8, CSF2, IL-1/IL1A) were generally down-regulated, and the intestinal inflammatory response was inhibited in the *G. elegans*-treated group, compared to the controls, which was similar to the results of the liver transcriptome results in the present study.

With respect to oxidative stress, there is increasing evidence that glycine enhances the intestinal mucosal barrier, reduces intestinal inflammation, and inhibits oxidative stress by inhibiting NF-κβ and TNF-α activation and IL-1 and IL-6 production [30]. In addition, both glycine and NAC protect the lipopolysaccharide (LPS)-induced intestinal barrier in piglets through the mTOR and MAPK signaling pathways [17,31,32]. MAPK signaling in mammals mainly includes MAPKs, extracellular signal-regulated kinase (ERK), c-Jun N-terminal kinase (JNK), and p38, which play important roles in cell growth, proliferation, differentiation, migration, inflammation, and survival [33,34,35,36]. P38 mitogen-activated protein kinases are activated primarily in response to inflammatory cytokines and cellular stress. In the ileum transcriptomes, the p38 gene was down-regulated in the *G. elegans*-treated group compared to the controls. It was speculated that *G. elegans* could reduce the activation of p38 in the MAPK signaling pathway by regulating glycine and NAC-related metabolism and could play a role in protecting intestinal cells from oxidative stress. In some pharmacological mechanism studies of *G. elegans*, koumine has been observed to significantly reduce phosphorylation of p38 and ERK in the LPS-mediated MAPK signaling pathway in mouse cells, all of which are consistent with the reduction in p38 activation [1].

### 4.4. G. elegans Regulates Lipid Metabolism

To explore the influence of *G. elegans* on other metabolic pathways, this study conducted a comprehensive network analysis based on the transcriptomics and metabolomics analyses results. In the MetScape analysis [37], we identified seven metabolic pathways directly related to lipids. These pathways include unsaturated fatty acid metabolism pathways: linoleic acid metabolism and arachidonic acid metabolism, saturated fatty acid metabolism, glycerolipid metabolism, sphingolipid metabolism, steroid hormone biosynthesis, and metabolic pathways. The liver is the main site of fatty acid synthesis in animals. It first synthesizes palmitate and then produces other saturated fatty acids and unsaturated fatty acids. Unsaturated fatty acids exist mainly in the form of phospholipids in cell membranes. Under the action of phospholipase, unsaturated fatty acids produce free arachidonic acid, which is then converted into leukotrienes or prostaglandins by lipoxygenase or cyclooxygenase. The balance of these two substances plays an important role in regulating lipid metabolism [38].

Additionally, glycosphingolipids are membrane components that can affect numerous cellular events, including homeostasis, adhesion growth, motility, apoptosis, proliferation, stress, and inflammatory responses [39]. Interestingly, the alteration of lipid metabolites was also reported to be associated with type 2 diabetes risk in metabolomics studies [40,41]. In the transcriptome studies, we found that PPAR signaling pathways are involved in the lipid metabolism regulation of *G. elegans* in pigs. PPAR ligands activate nuclear hormone receptor family receptors, control many cell metabolism pathways, and play an important role in regulating cell differentiation, growth, and metabolism in higher organisms [42]. Three subtypes, namely PPAR alpha, PPAR beta, and PPAR-gamma, have been reported. PPAR alpha is expressed in the liver, kidney, heart, muscle, fat, and other organs [43] and is involved in the fatty acid metabolism and lipid transport in the liver, in lipoprotein oxidation and combination, and the uptake of fatty acids and assembly [44], and plays an important role in regulate hepatic fat metabolism. DEGs, including CPT-1, PCK1, and PATP, were enriched in the PPAR signaling pathway, andCPT-1 and PCK1 are downstream genes regulated by PPAR.

CPT-1 expression was increased by *G. elegans* supplementation. CPT-1 is a mitochondrial enzyme that plays an important role in regulating fatty acid metabolism. Lipid consumption is mainly oxidized by FAO through transport to the mitochondrial matrix, and the transport process is mediated by the CPT system, which is composed of CPT-1, acylcarnitine translocation enzyme, and CPT2 [45]. The activation of CPT-1 in an obesity (DIO) model has been reported to increase energy utilization and fatty acid oxidation [46]. Additionally, according to the RNA-seq results, the expression of CPT-1 was increased, and it was speculated that *G. elegans* could regulate the lipid metabolism process through CPT-1, thus improving the hepatic function. PCK1 catalyzes gluconeogenesis, that is, the synthesis of glucose, and plays an important role in maintaining glucose homeostasis. By regulating the expression of this gene, blood glucose levels can be maintained within a well-defined range. According to the report, the excessive expression of PCK1 can result in type II diabetes symptoms [47], and excessive sugar dysplasia may also lead to the occurrence of metabolic diseases such as insulin resistance and hyperglycemia, indicating that PCK1 is important in glucose homeostasis. In the present study, the PPAR pathway inhibited the expression of PCK1, suggesting that *G. elegans* could be used to inhibit the excessive production of glycogen via PCK1-mediated regulation of sugar dysplasia, thus improving metabolic diseases such as type II diabetes.

## 5. Conclusions

The addition of 20 g/kg *G. elegans* powder to feed is nontoxic to pigs. *G. elegans* mediates the MAPK signaling pathway through glycine and NAC metabolism regulation-related gene expression, reduces p38 activation, and exerts antioxidant and anti-inflammatory effects. The PPAR signaling pathway mediates lipid metabolism, including the expression of important genes such as PCK1 and CPT-1, to regulate hepatic lipid metabolism and gluconeogenesis and improve hepatic function. Collectively, results of the current study provide a clearer understanding of the molecular mechanism of the pharmacological effects of *G. elegans*, which is of great significance for a safer and more rational application of this herbal medicine.

## Figures and Tables

**Table 1:** Effect of *G. elegans* powder on the growth performance of piglets.

Items	Treatment		SEM ^1^	*p*-Value ^2^
	Control	*G. elegans*-Treated		L
Num	10	30	-	-
Average feed intake, kg	13.58	9.02	0.165	<0.001
Average gain, kg	5.32	4.06	0.038	<0.001
Feed conversion ratio	2.55	2.22	1.176	<0.001

^1^ SEM = Standard error of mean. ^2^ L = Linear effect of *G. elegans*.

**Table 2:** Serum chemical parameters of the control group and the *G. elegans*-treated group.

Items	Treatment		*p*-Value
	Control	*G. elegans*-Treated	
WBC, 10^9^/L	18.13	25.22	0.040
LYMPH, 10^9^/L	8.51	12.13	0.023
EOS, 10^9^/L	1.14	1.76	0.058
LYMPH, %	47.01	48.63	0.304
EOS, %	8.07	7.07	0.667
RBC, 10^12^/L	7.919	9.188	0.852
HGB, g/L	122.33	140	0.504
MCV, fL	81.75	78.58	0.027
MCH, pg	16.3	18.7	0.611
MCHC, g/L	202	236.5	0.287
RDW_CV, %	16.09	16.62	0.413
RDW_SD, fL	52.6	52.1	0.077
HCT, %	64.82	71.77	0.163
PLT, 10^9^/L	256.2	984	0.785
MPV, fL	10.56	11.23	0.096
PDW	14.24	15.08	0.106
PCT, %	0.273	1.111	0.186

WBC (white blood cell count), LYMPH (lymphocyte count), EOS (eosinophil count), RBC (red blood cell count), HGB (hemoglobin concentration), MCV (mean corpuscular volume), MCH (mean corpuscular hemoglobin), MCHC (mean corpuscular hemoglobin concentration), RDW (red cell distribution width), HCT (hematocrits), PLT (platelet count), MPV (mean platelet volume), PDW (platelet distribution width), PCT (plateletcrit).

**Table 3:** Results of quantitative qRT-PCR validation.

Gene Symbol	Tissue	RNA-Seq		qRT-PCR	
		*p*-Value	Fold Change	*p*-Value	Fold Change
Atp2b3	Liver	<0.001	3.57	0.014	2.39
Mat2a	Liver	<0.001	2.18	0.008	1.70
Chdh	Liver	0.026	2.35	0.024	1.48
Slc20a2	Ileum	0.006	2.05	0.010	1.89
Slc28a1	Ileum	0.008	2.02	0.028	2.02
Mt3	Ileum	<0.001	3.49	0.008	3.75

**Table 4:** The positive ion pattern of differentially expressed metabolites with VIP > 1 and *p* < 0.05 in the plasma of piglets exposed to *G. elegans* based on ANOVA.

Metabolites	VIP	log2(Fold Change)	*p*-Value
Methylsulfonylmethane	2.24	2.44	<0.001
3-(4-Hydroxy-5-oxo-3-phenyl-2,5-dihydro-2-furanyl) propanoic acid	1.46	1.58	<0.001
Visnagin	1.53	1.66	<0.001
Beta-Naphthoxyacetic Acid	1.57	1.70	<0.001
Menadiol	1.41	1.52	<0.001
N-Feruloylserotonin	2.29	2.52	<0.001
Dibenzo-1,4-dioxin	1.45	1.55	0.001
(-)-Akuammicine	1.98	2.20	0.001
1-Naphthol	1.38	1.51	0.002
4-Methylene-2-oxoglutarate	1.45	1.58	0.002
5-Formyl-2-furoic acid	1.45	1.60	0.003
N-Acetyl-L-phenylalanine	1.66	1.75	0.003
Menadione	1.18	1.32	0.005
Phenylacetylglycine	1.18	1.25	0.006
Indole-3-carbidol	1.47	1.53	0.010
Furathiazole	1.32	1.39	0.016
1-Stearoyl-2-arachidonoyl-sn-glycero-3-phosphoserine	1.44	1.72	0.023
Spirodiclofen	2.35	2.35	0.028
Pelargonidin	1.19	−1.20	0.030
2,5-Dimethyl-4-ethoxy-3(2H)-furanone	1.11	1.35	0.041
7-alpha-Hydroxy-3-oxochol-4-en-24-oic acid	1.31	1.47	0.047

**Table 5:** The results of differentially expressed metabolites in negative ion mode with VIP > 1 and *p* < 0.05 in plasma of piglets exposed to *G. elegans* based on ANOVA.

Metabolites	VIP	log2(Fold Change)	*p*-Value
Maslinic acid	2.54	2.19	0.001
10-Acetyl-9-hydroxy-7,7-dimethyl-2,6,6a,7,11a,11b-hexahydro-11H-pyrrolo[1′,2′:2,3]isoindolo[4,5,6-cd]indol-11-one	2.23	1.99	0.003
Adipic acid	2.17	1.94	0.004
4-[(2-Isopropyl-5-methylcyclohexyl)oxy]-4-oxobutanoic acid	1.14	−0.90	0.008
Glycine	1.23	1.02	0.009
Imazamethabenz	1.39	1.13	0.009
p-Tolyl beta-d-glucuronide	1.50	1.23	0.011
Phenylacetylglycine	1.45	1.18	0.011
(+)-CP 55,940	1.71	1.42	0.011
d-(-)-Quinic acid	2.08	1.89	0.013
(-)-CP 55,940	2.00	1.65	0.013
Cascarillin	1.07	−0.81	0.020
Picrasin C	1.14	−0.84	0.026
10-[4-(2,4,4-Trimethyl-2-pentanyl) phenoxy]-1-decanol	2.08	−1.48	0.038
N-Acetyl-L-cysteine	1.06	0.95	0.041
4-Methylphenol	1.47	1.16	0.043
Actinoquinol	1.11	−0.81	0.046
Glycol stearate	2.35	−1.79	0.49

## Data Availability

The data used to support the findings of this study are available from the corresponding author upon reasonable request.

## Abbreviations

BP	biological processes
CD	Compound Discoverer
DEGs	differentially expressed genes
ERK	extracellular signal-regulated kinase
*G. elegans*	*Gelsemium elegans*
GO	genetic ontology
JNK	c-Jun N-terminal kinase
KEGG	Kyoto Encyclopedia of Genes and Genomes
LPS	lipopolysaccharide
MF	molecular function
MAPKs	mitogen-activated protein kinases
NAC	N-acetylcysteine
NAFLD	Nonalcoholic fatty liver disease
PCA	principal component analysis
PLS-DA	partial least squares-discriminant analysis
